# Spatial analysis of bladder, kidney, and pancreatic cancer on upper Cape Cod: an application of generalized additive models to case-control data

**DOI:** 10.1186/1476-069X-8-3

**Published:** 2009-02-10

**Authors:** Verónica Vieira, Thomas Webster, Janice Weinberg, Ann Aschengrau

**Affiliations:** 1 Department of Environmental Health, Boston University School of Public Health, 715 Albany Street, Boston, MA 02118, USA; 2 Department of Biostatistics, Boston University School of Public Health, 715 Albany Street, Boston, MA 02118, USA; 3 Department of Epidemiology, Boston University School of Public Health, 715 Albany Street, Boston, MA 02118, USA

## Abstract

**Background:**

In 1988, elevated cancer incidence in upper Cape Cod, Massachusetts prompted a large epidemiological study of nine cancers to investigate possible environmental risk factors. Positive associations were observed, but explained only a portion of the excess cancer incidence. This case-control study provided detailed information on individual-level covariates and residential history that can be spatially analyzed using generalized additive models (GAMs) and geographical information systems (GIS).

**Methods:**

We investigated the association between residence and bladder, kidney, and pancreatic cancer on upper Cape Cod. We estimated adjusted odds ratios using GAMs, smoothing on location. A 40-year residential history allowed for latency restrictions. We mapped spatially continuous odds ratios using GIS and identified statistically significant clusters using permutation tests.

**Results:**

Maps of bladder cancer are essentially flat ignoring latency, but show a statistically significant hot spot near known Massachusetts Military Reservation (MMR) groundwater plumes when 15 years latency is assumed. The kidney cancer map shows significantly increased ORs in the south of the study area and decreased ORs in the north.

**Conclusion:**

Spatial epidemiology using individual level data from population-based studies addresses many methodological criticisms of cluster studies and generates new exposure hypotheses. Our results provide evidence for spatial clustering of bladder cancer near MMR plumes that suggest further investigation using detailed exposure modeling.

## Background

**Figure 1:**
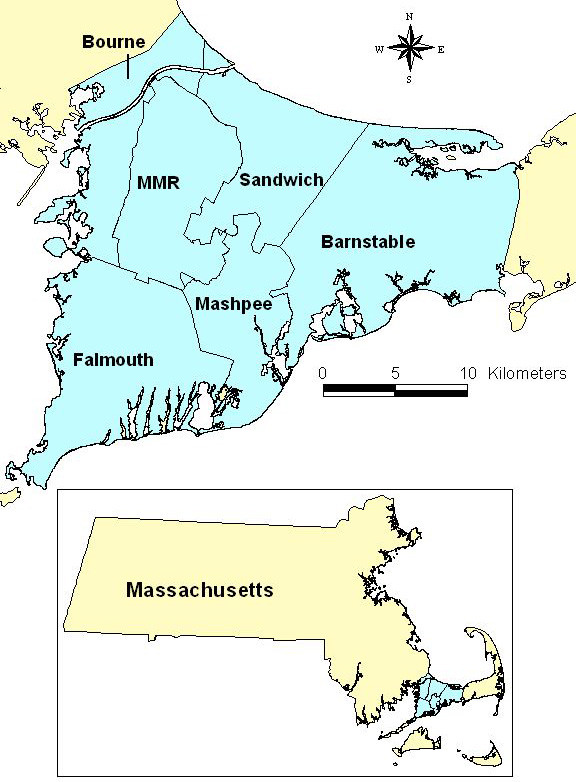
**Upper Cape Cod study area**. Cape Cod is located in Massachusetts in the northeast United States.

In 1988, elevated cancer incidence in the upper Cape Cod region of Massachusetts (Figure 1) prompted a large epidemiological study of all cancers to investigate possible environmental risk factors, including air and water pollution associated with the Massachusetts Military Reservation (MMR), pesticide applications to cranberry bogs, particulate air pollution from a large electric power plant, and tetrachloroethylene-contaminated drinking water from vinyl-lined asbestos cement distribution pipes [1-11]. Positive associations were observed, but environmental exposures explained only a portion of the excess cancer incidence. This population-based case-control study provides information on individual-level covariates and residential history useful for a secondary spatial analysis.

Methods for mapping point-based epidemiologic data have received less attention than mapping areal data [12]. Generalized additive models (GAMs), a type of statistical model that combines smoothing with the ability to analyze binary outcome data and adjust for covariates, provide a useful framework for examining point data [13-18]. Using individual-level information and location in a generalized additive model, we calculated the crude and adjusted odds ratios for bladder, kidney, and pancreatic cancers on upper Cape Cod. These analyses, unlike many registry-based maps, have the advantage of controlling for spatial confounders, examining the effect of latency, and allowing for hypothesis testing for the significance of location in the disease maps. The objectives of the present analyses are to identify exposure hypotheses for further investigation and to demonstrate spatial epidemiology using generalized additive models.

## Methods

### Study Population

We investigated the association between residence and kidney, pancreatic, and bladder cancer on upper Cape Cod, Massachusetts using data from a population-based case-control study [1]. Spatial analyses of breast, lung, and colorectal cancer were previously reported in Vieira et al. [17]. The Massachusetts Cancer Registry was used to identify incident cancer cases diagnosed from 1983–1986. Participants were restricted to permanent residents (living on upper Cape Cod at least 6 months of the year) with complete residential histories. A total of 62 bladder cancer cases, 35 kidney cancer cases, and 37 pancreatic cancer cases were included.

There were 885 bladder cancer controls, 803 kidney cancer controls, and 651 pancreatic cancer controls. A large number of controls were available for the present analyses because the parent study investigated nine cancer types including breast, lung, and colorectal cancers with larger numbers of cases [1]. See earlier papers [2,3] for a detailed description of the methods used to define the study population, including the rationale for the method of control selection. Briefly, controls were chosen to represent the underlying population that gave rise to all the cancer cases from the large epidemiologic study, i.e., permanent residents of upper Cape Cod during the same time period. Controls were frequency matched to all cancer cases on age, gender, and vital status. It is important to note that controls were not matched on town of residence. Because many of the cases were deceased or elderly, three different sources of controls were used: (1) random digit dialing for living controls less than 65 years of age; (2) Centers for Medicare and Medicaid Services (formerly the Health Care Financing Administration) for living controls 65 years of age or older; and (3) death certificates for controls who had died from 1983–1986.

Participants or their next-of-kin completed an extensive interview, providing information on demographics (age, sex, marital status, and education), a forty-year residential history, and potential confounders. "Index years" were randomly assigned to controls in a distribution similar to that of diagnosis years for cases. We used the diagnosis and index years to estimate timing and duration of environmental exposure among case and controls, respectively. The Institutional Review Board of Boston University Medical Center approved the research.

### Geographical Information System (GIS)

All residential addresses reported by participants in the upper Cape Cod area over the forty-year period prior to the diagnosis or index year were eligible for the spatial analysis. Many participants lived at more than one address during their residential history on upper Cape Cod. We excluded all addresses where residency time began after the diagnosis date for cases and index date for controls. The bladder cancer data set included 95 case locations and 1,382 control locations. The kidney cancer data set included 54 case locations and 1,220 control locations. The pancreatic cancer data set included 49 case locations and 1,005 control locations.

**Figure 2:**
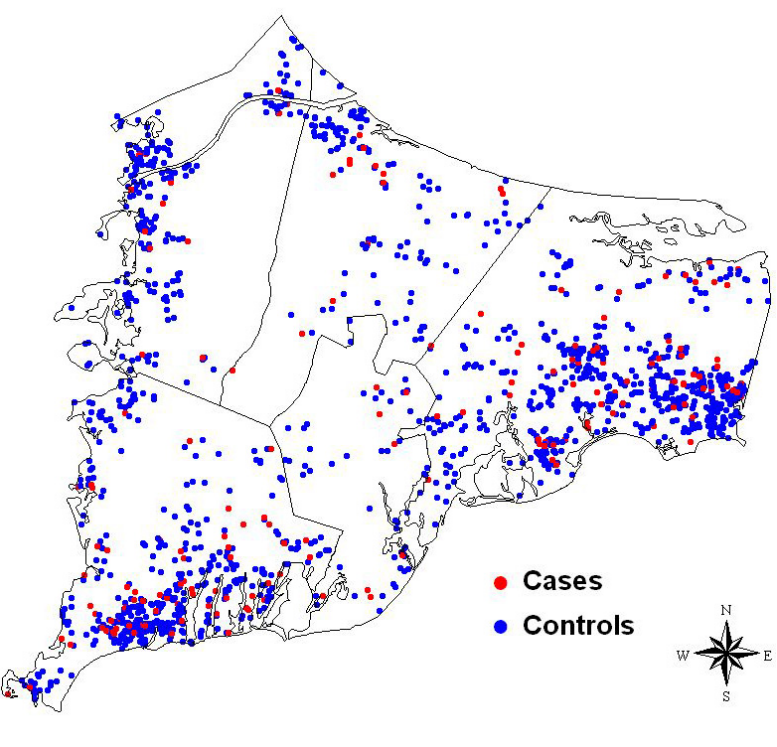
**Spatial distribution of participants**. Each point represents the residence of one participant. Locations have been geographically altered to preserve confidentiality.

Locations of participant residences were geocoded using the Massachusetts State Plane Coordinate System with North American Datum 1983 (NAD1983). Geocoding, the process where map coordinates are assigned to each street address, was done without knowledge of case status, and the final data were checked for accuracy. GIS allows us to map the coordinates of participants and link the location to individual interview data and environmental information. Figure 2 shows the distribution of bladder, kidney, and pancreatic cancer cases and controls in the study area. The sparse population in the center of the study area is where the Massachusetts Military Reservation is located (Figure 1). To preserve confidentiality, the figure was created by randomly placing residences within a small grid that includes the actual location. Actual locations were used in the analysis.

### Generalized Additive Models (GAMs)

We estimated local disease odds using generalized additive models, a form of non-parametric or semi-parametric regression with the ability to analyze binary and continuous outcome data while adjusting for covariates [13,18]. We modeled location, a potential surrogate measure of exposure, using a bivariate smooth (S) of spatial coordinates (x_1_) and (x_2_)

(1) logit [p(x_1_, x_2_)] = S(x_1_, x_2_) + **γ'z**

where the left-hand side is the log of the disease odds at location (x_1_, x_2_), **z **is a vector of covariates, and γ is a vector of parameters. Throughout this paper, we will refer to (x_1_) and (x_2_) as longitude and latitude, although (x_1_) and (x_2_) are technically measures of distance and not degrees. The model is semiparametric because it has the nonparametric smooth but the covariates are modeled parametrically. Without the smooth function, S(x_1_, x_2_), the model becomes an ordinary logistic regression on the covariates.

Spatial confounding occurs when risk factors for a disease are not evenly distributed. For example, a cluster of lung cancer may be due to an increased density of smokers. A group of core confounders, chosen *a priori *based on the current scientific literature or study design, was included in all adjusted cancer analyses: vital status at interview, gender, race, age at diagnosis or index year, and usual number of cigarettes smoked. In the kidney and bladder cancer analyses, we also controlled for history of urinary tract infection or stone. In the pancreatic cancer analysis, we also controlled for usual alcohol consumption. We dropped other covariates from the model because they did not change the appearance of the map, including education level, prior medical treatment with irradiation, and occupational exposure to solvents. The covariate with the largest percentage of missing data was education (7% of the participants). Since education was not a confounder in the final model, we did not exclude these participants.

We used a loess smooth which adapts to changes in population density [13]. The amount of smoothing, referred to as the span size, depends on the percentage of the data points in the neighborhood. As a result, the geographic extent of the neighborhood is smaller in densely populated areas and larger in areas with sparse population. We determined the optimal amount of smoothing for each map by minimizing the Akaike's Information Criterion (AIC). Small span sizes produce bumpier surfaces and larger span sizes produce smoother surfaces. As the span size increases, the amount of bias in the fit increases and the variance decreases [13]. We created a rectangular grid covering the study area using the minimum and maximum longitude and latitude from the study subjects (The GAM does not predict locations beyond where the subjects live). Grid points lying outside the outline map of the study area were clipped, as were areas where people cannot reside (e.g., ocean or conservation land).

We converted from log odds to odds ratios (ORs) using the odds of disease in the whole study area as the reference. When controls are appropriately sampled from the population giving rise to the cases, the case-control ratio (disease odds) in a subset of the area should be proportional to the disease incidence rate and the odds ratio estimates rate ratio. In order to make maps visually comparable, we mapped all results using the same dark blue to dark red continuous (unclassified) color scale and range of odds ratios, 0.25–2.50. This range covers most but not all of the ORs observed in our analyses, preventing maps from being washed out by an area of extremely high or low ORs. We determined the presence of spatial confounding by visually comparing crude and adjusted maps. If their optimal span sizes differ, we also compared maps using a common span size, allowing us to distinguish between changes due to confounder adjustment and changes due to span size.

GAMs also provide a framework for hypothesis testing. We first tested the null hypothesis that case status does not depend on the smooth term using a permutation test based on the difference of the deviances of model (1) with and without the smooth term. We condition on the number of cases and controls, preserving the relationship between case/control status and covariates, and randomly assign individuals to locations. We carry out 999 permutations of location in addition to the original. For each permutation, we ran the GAM using the optimal span of the original data and computed the deviance statistic. We do this to preserve the spatial resolution of the original map; the test is thus conditional on the span size. We divide the rank of the observed value by 1000 to obtain a p-value. We used a p-value cut off of 0.05 as a screening tool for possibly meaningful associations. We discuss results as "significant" if the associated p-values are less than 0.05, but acknowledge that some results may be due to chance.

If the global deviance test indicates that the map is unlikely to be flat, we next want to locate areas of the map that exhibit unusually high or low disease odds. We examined point-wise departures from the null hypothesis using permutation tests if the global statistic indicated that location was significant at the 0.05 level. We obtained a distribution of the log odds at every point using the same set of permutations we used for calculating the global statistics. We defined areas of significantly decreased odds ("cold spots") to include all points that ranked in the lower 2.5% of the point-wise permutation distributions and areas of elevated odds ("hot spots") to include all points that ranked in the upper 2.5% of the point-wise permutation distributions. By drawing the 2.5% and 97.5% contour lines, we mapped areas of significantly decreased and increased risk.

Webster et al. [16] provides a detailed discussion of the statistical methods, analyses using synthetic data, and a comparison with the kernel method of Kelsall and Diggle [14]. We used S-Plus [19] to perform the generalized additive modeling and ArcGIS [20] to map the results of our analyses. Sample program code is available at http://www.cireeh.org/pmwiki.php/Main/SpatialEpidemiology.

### Residential History

Our initial, no-latency analyses included all eligible residences with complete address information to allow for geocoding. Therefore, exposures occurring up to diagnosis were assumed to contribute to the risk of disease. By including all addresses, the disease outcome is replicated with the same covariates but different residence for each participant. However, solid cancers initiated by exposure to carcinogens typically take more than a decade to develop. For cancers with sufficient case numbers, we performed a fifteen-year latency analysis by restricting inclusion to the residences occupied by participants at least fifteen years prior to the diagnosis or index year (Residences within the fifteen year window were excluded because geographical location within that window was assumed not relevant to the outcome). Because the inclusion of multiple residences for the same individual may bias our statistical model, we also performed an analysis of residence of longest duration.

## Results

Participants were predominantly white and over 60 years of age (Table 1). Cases were more likely to be smokers. A larger proportion of bladder and kidney cancer cases than controls were male and less educated. Pancreatic cancer cases and controls were predominantly female. Prior medical radiation was more common among pancreatic cases than controls, and history of urinary tract infection or stone was more common among bladder and kidney cancer cases than controls.

**Table 1:** Distribution (%) of Selected Characteristics of Cases and Controls

	Bladder Cancer		Kidney Cancer		Pancreatic Cancer	
Characteristic	Cases (n = 62)	Controls (n = 885)	Cases (n = 35)	Controls (n = 803)	Cases (n = 37)	Controls (n = 651)
Gender						
Male	72.6	52.5	62.9	48.6	35.1	38.2
Female	27.4	47.5	37.1	51.4	64.9	61.8
Race						
White	98.4	96.4	94.3	96.8	97.3	96.9
Other	1.6	3.6	5.7	3.2	2.7	3.1
Age at diagnosis or index year (y)						
1–49	0.0	1.5	2.9	1.2	0.0	0.0
50–59	9.7	9.7	8.6	7.0	5.4	2.2
60–69	40.3	39.6	28.6	42.3	13.5	33.0
70–79	37.1	34.4	51.4	40.7	59.5	46.2
80+	12.9	14.8	8.5	8.8	21.6	18.6
Education level (y)						
Less than 12	31.0	19.2	20.6	17.3	13.9	19.7
12	25.9	32.4	32.4	34.6	33.3	37.1
13–15	13.8	24.6	20.6	25.9	22.2	21.4
16 or more	29.3	23.8	26.5	22.2	30.6	21.8
Ever regular cigarette smoker^a^	88.7	66.3	74.3	66.7	51.4	35.0
Ever regular alcohol drinker^a^	----^b^	----^b^	----^b^	----^b^	73.0	83.3
Ever exposed occupationally to solvents^c^	33.9	25.8	25.7	25.2	8.1	21.2
History of urinary tract infection or stone	64.5	27.9	54.3	28.8	----^b^	----^b^
Prior medical treatment with radiation	19.4	13.1	11.4	13.4	83.8	14.1
Alive at interview	66.1	56.0	51.4	60.4	5.4	35.0

^a ^Cigarette smoking was modeled as a categorical variable for usual number of cigarettes smoked. Alcohol was modeled as a dichotomous variable for heavy drinker.

^b ^Not a risk factor for this cancer type.

^c ^Based on answers to direct questions regarding exposure to solvents and degreasers, benzene, and paint thinners.

### Bladder Cancer

**Figure 3:**
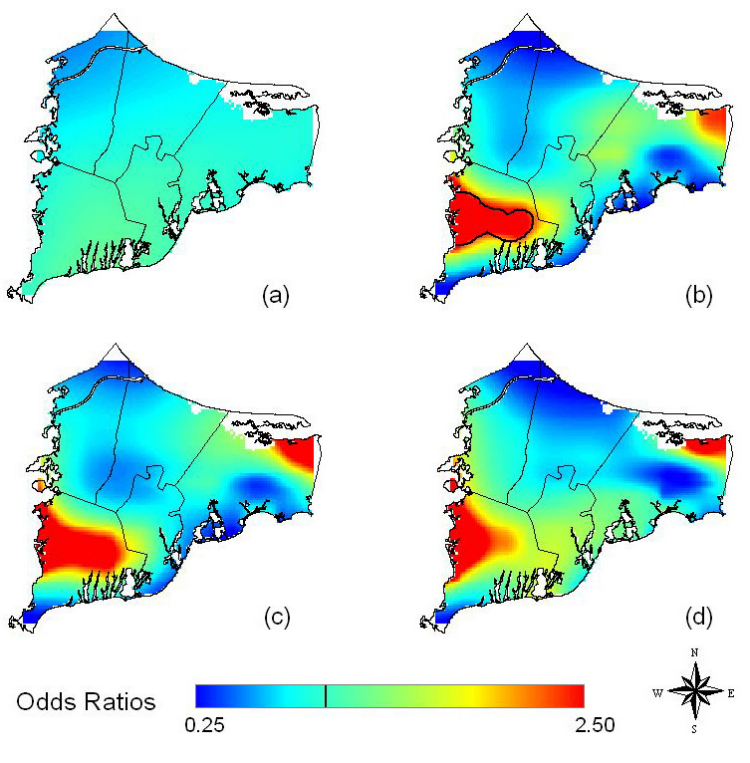
**Bladder cancer results**. Odds ratios are relative to the whole study area. a) Adjusted, no latency. b) Adjusted, 15 years of latency. Assuming 15 years of latency increases the magnitude of hot and cold spots. Black contour lines denote areas of significantly increased and decreased risk at the 0.05 level. c) Crude, 15 years of latency, created using the optimal span (0.30) of the adjusted map. Little difference from the adjusted map suggests spatial confounding is not an issue. d) Adjusted, 15 years of latency. Restriction to residences of longest duration has little effect when the same span (0.30) is used as for all residences.

Assuming no latency, location was not statistically significant at the 0.05 level in the crude (not shown) and adjusted maps (Table 2 and Figure 3a). When we assumed 15 years of latency, the adjusted analysis (Figure 3b) was significantly different from flat with an optimal span size of 0.30. Black contour lines denote areas of significantly increased and decreased risk at the 0.05 level. The point-wise tests of significance showed an area of significantly increased odds ratios in the southwest town of Falmouth. Disease odds in certain areas were six times higher than the study area as a whole. The adjusted and crude analysis (Figure 3c) were similar when the same span size was used, suggesting spatial confounding was not an issue.

**Table 2:** Summary of bladder cancer models showing degree of smoothing and global test statistic

Analysis	Latency (yrs)	Span^a^	Cases/Controls^c^	Deviance p-value^d^	Figure #
Crude All Residences	0	0.90	95/1382	0.57	--
Adjusted All Residences	0	0.90	95/1382	0.36	3a
Adjusted All Residences	15	0.30	45/665	0.05	3b
Crude All Residences	15	0.40	45/665	0.11	--
Crude All Residences	15	0.30^b^	45/665	-----^e^	3c
Adjusted Longest Duration Residence	15	0.30^b^	29/417	-----^e^	3d
Adjusted Longest Duration Residence	15	0.95	29/417	0.41	--

^ Span is the percentage of data used for smoothing the model.^

^a ^Optimal span obtained by using the Akaike's Information Criterion (AIC) unless otherwise noted.

^b ^Same span as for adjusted, 15 years latency, all residences.

^c ^Number of residences contributed to the analysis by participant status.

^d ^Null hypothesis is that the map is flat.

^e ^The p-values were omitted because the maps were created using a non-optimal span.

We also restricted the adjusted 15-year latency analysis to residences of longest duration. Using the same span size as before (0.30), Figure 3d shows that although cluster size and shape changed, the overall spatial pattern remained similar, suggesting that the use of multiple residences did not cause bias. The optimal span for the longest duration analysis was 0.95, likely due to the reduced sample size; using the larger span size resulted in a smoother surface (not shown).

### Kidney Cancer

**Figure 4:**
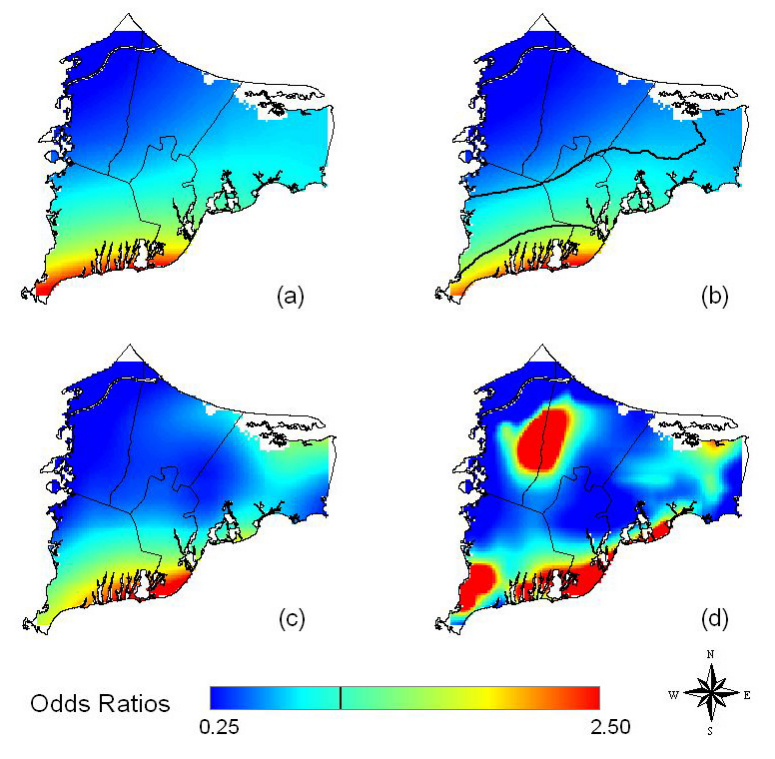
**Kidney cancer results**. Odds ratios are relative to the whole study area. a) Crude, no latency, created using the optimal span (0.90) of the adjusted map. b) Adjusted, no latency, optimal span. Black contour lines denote areas of significantly increased and decreased risk at the 0.05 level. Lack of important differences between the crude and adjusted maps suggests spatial confounding is not an issue. c) Adjusted, no latency, span of 0.40. Results are similar to optimal span. d) Adjusted, no latency, span of 0.15. Small span size results in more spatial variation in risk.

The crude and adjusted analyses predicted similar results (Table 3 and Figures 4a, b) when the same span size was used, suggesting spatial confounding was not an issue. The optimal span size for the adjusted analysis was 0.90. The map for kidney cancer shows a sloped surface with significantly increased odds ratios in the south of the study area and decreased odds ratios in the north. As suggested by these maps, the combination of a large optimal span and statistical significance indicates that the risk surface is approximated by a tilted plane. We restricted participants' addresses to the one of longest duration and observed a similar tilted plane. We did not have sufficient cases to examine latency.

**Table 3:** Summary of kidney cancer models showing degree of smoothing and global test statistic

Analysis	Latency (yrs)	Span^a^	Cases/Controls^d^	Deviance p-value^e^	Figure #
Crude All Residences	0	0.95	54/1220	<0.001	--
Crude All Residences	0	0.90^b^	54/1220	-----^f^	4a
Adjusted All Residences	0	0.90	54/1220	<0.001	4b
Adjusted All Residences	0	0.40^c^	54/1220	-----^f^	4c
Adjusted All Residences	0	0.15^c^	54/1220	-----^f^	4d

Span is the percentage of data used for smoothing the model.

^a ^Optimal span obtained by using the Akaike's Information Criterion (AIC) unless otherwise noted.

^b ^Same span as for adjusted, all residences.

^c ^Local minimum span was used.

^d ^Number of residences contributed to the analysis by participant status.

^e ^Null hypothesis is that the map is flat.

^f ^The p-values were omitted because the maps were created using a non-optimal span.

The AIC curve for the adjusted kidney cancer model indicates two local minima at span sizes of 0.15 and 0.40 before reaching the global minimum (and optimal span) of 0.90. We repeated the adjusted kidney analysis using spans of 0.40 (Figure 4c) and 0.15 (Figure 4d). The map for span of 0.40 is similar to the results we obtained using the optimal span. The small span of 0.15 produced a bumpier surface, including an area of highly increased risk in the center of the study region. This hot spot is likely spurious due to the sparse population in this area (Figure 2). We did not test for statistical significance of location in models that did not use the optimal span size.

### Pancreatic Cancer

**Figure 5:**
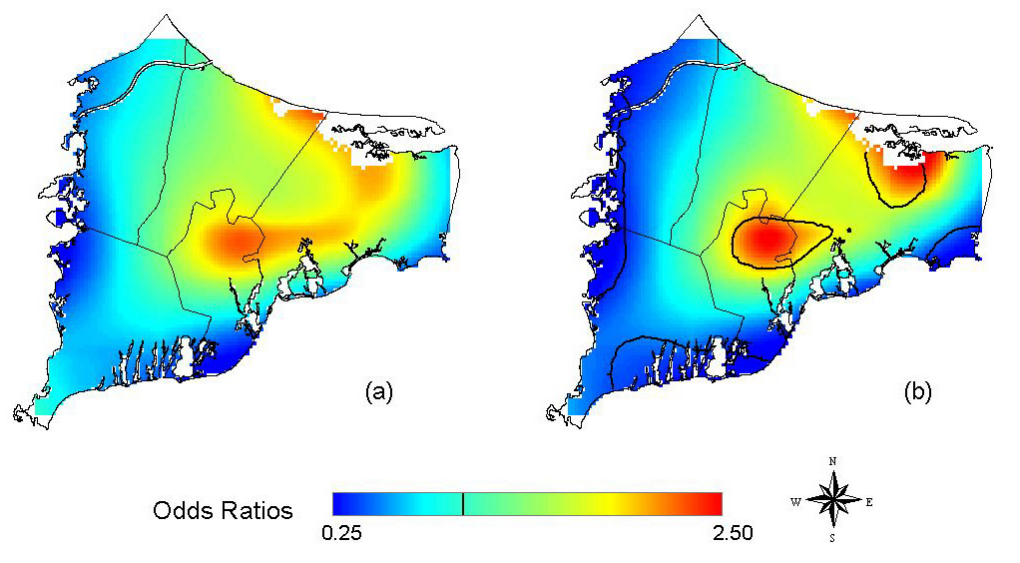
**Pancreatic cancer results**. Odds ratios are relative to the whole study area. a) Crude, no latency, created using the optimal span (0.40) of the adjusted map. b) Adjusted, no latency, optimal span. Black contour lines denote areas of significantly increased and decreased risk at the 0.05 level. Difference of the crude and adjusted maps indicates spatial confounding.

The variation of risk was smaller in the crude analysis (Table 4 and Figure 5a) compared to the adjusted analysis (Figure 5b) when the same span size was used. Thus, spatial confounding was partially masking differences in the crude analysis. By mapping the model results with individual confounders, we determined alcohol use was the single most important variable responsible for this difference. The point-wise tests of significance showed a hot spot in northern Mashpee and another in northern Barnstable.

**Table 4:** Summary of pancreatic cancer models showing degree of smoothing and global test statistic

Analysis	Latency (yrs)	Span^a^	Cases/Controls^c^	Deviance p-value^d^	Figure #
Crude All Residences	0	0.75	49/1005	0.04	--
Crude All Residences	0	0.40^b^	49/1005	-----^e^	5a
Adjusted All Residences	0	0.40	49/1005	0.02	5b

Span is the percentage of data used for smoothing the model.

^a ^Optimal span obtained by using the Akaike's Information Criterion (AIC) unless otherwise noted.

^b ^Same span as for adjusted, all residences.

^c ^Number of residences contributed to the analysis by participant status.

^d ^Null hypothesis is that the map is flat.

^e ^The p-values were omitted because the maps were created using a non-optimal span.

We examined potential bias from multiple residences in the pancreatic cancer analysis by restricting participants' addresses to the one of longest duration. The spatial patterns of the resulting map differ from the map with all residences suggesting a bias may exist (not shown). The cluster in the center of the study area (Figure 5b) is no longer elevated. The case locations that contributed to the cluster in the analysis with all residences were for different individuals, but were of shorter residency duration than other residences. We were unable to consider latency because there were too few cases.

## Discussion

In our analyses, bladder cancer on upper Cape Cod displayed an area of increased risk when we considered latency, and location became a significant predictor. Many geographic analyses based on cancer registry data only use address at diagnosis. The greater spatial variation in bladder cancer with increased latency is consistent with misclassification of geographically associated risk factors, including environmental exposures. If population movement is random with respect to disease status, ignoring latency should increase nondifferential exposure misclassification and tend to make maps flatter.

Results of the kidney cancer analysis indicated that there was latitudinal variation causing the odds ratios to tilt significantly in magnitude from north to south. The AIC curve resulted in two additional minima of 0.15 and 0.40. Choice of span size is one of the most important issues in smoothing [13]. We used the Akaike Information Criterion, a computationally feasible method for choosing an "optimal" span based on the tradeoff between bias and variance of the smooth. There are, however, problems with automatic selection procedures. Selecting the span that optimizes the bias-variance tradeoff is not necessarily the same as understanding the importance of map features. Rather than using a single span, there may be important aspects of the data at different scales. New methods are needed to address this issue, e.g., [21].

The adjusted pancreatic cancer map showed more pronounced hot and cold spots compared to the crude analysis. Rather than creating disease clusters as is often assumed, spatial confounding was partially hiding areas of increased risk. Unlike cancer registry maps which contain limited data on covariates, our analyses controlled for many covariates available in the case-control study questionnaire.

A number of epidemiologic studies have examined cancer and environmental exposures on Cape Cod [1-11]. Previous studies investigated the association between kidney, pancreatic and bladder cancer and tetrachloroethylene (PCE) in drinking water from vinyl-lined asbestos cement distribution pipes [2,3] and cranberry cultivation [5]. Increased relative risks were found for bladder and pancreatic cancer in the highest PCE-exposed individuals. No evidence was found for increased risks of bladder, kidney, and pancreatic cancer associated with living within 2600 ft of cranberry bogs. Adding PCE exposure and proximity to cranberry bogs to the current spatial models had no effect on the appearance of the maps.

**Figure 6:**
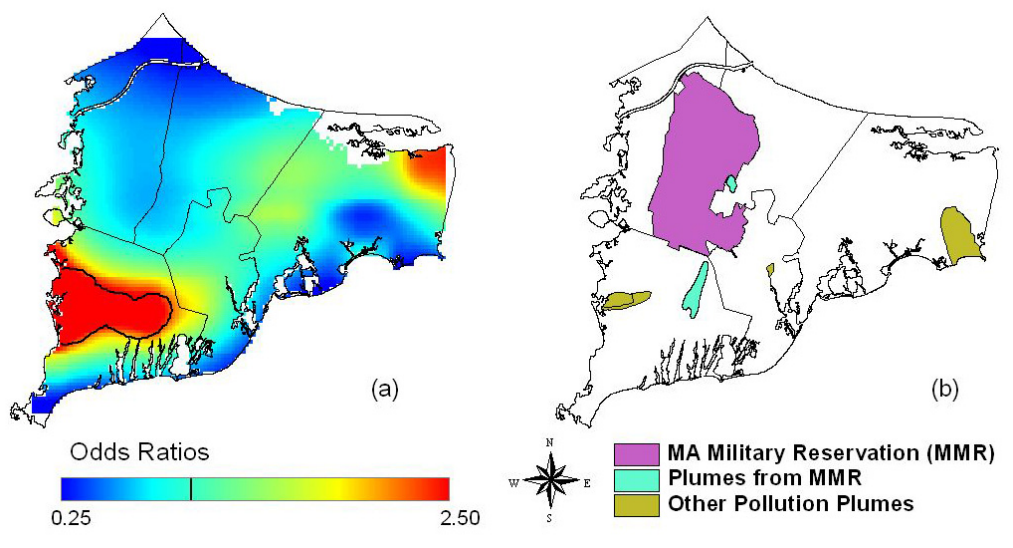
**Groundwater plumes, the Massachusetts Military Reservation (MMR), and significant bladder cancer hot spots**. a) Bladder cancer map (Fig. 3b), adjusted, 15 years of latency with an optimal span of 0.30. Odds ratios are relative to the whole study area. b) Location of the MMR and groundwater plumes from the MMR and other sources including landfills.

Our analysis located a significant bladder cancer "hot spot" to the southwest of the Massachusetts Military Reservation (MMR, Figure 6a). We also found a significant pancreatic cancer hot spot near the MMR when using all residences, but this area was no longer elevated when the analysis was restricted to residence of longest duration. Earlier research had found a modest increased risk of pancreatic cancer within 3 km of gun and mortar training sites on the military base [4]. French and Wand [11] reported an area of increased risk for breast cancer southeast of the MMR. In a prior spatial analysis, we also found a significant breast cancer hot spot near the MMR [17]. Others have found suggestions of a link between low birth weight and proximity to the base [22].

Overlaying maps of odds ratios with maps of pollution sources can generate hypotheses about exposure. Caution is needed, however, because many geographic features overlap. The Massachusetts online repository of geographically coded features for shape files potentially related to environmental exposure [23] was explored to generate hypotheses for further investigation. Groundwater plumes were of particular interest because of our earlier analyses of breast cancer and pollution of drinking water [24]. With no prior knowledge of any geographic relationship to bladder cancer, we compared the two data sets (Figure 6), and found a suggestive overlap between the bladder cancer hot spots and ground water plumes, some from the MMR and others from landfills and wastewater treatment facilities. Groundwater plumes in the Barnstable and Falmouth areas are currently being modeled to investigate further this hypothesis.

Case-control studies are one of the standard epidemiologic tools for investigating associations between disease and exposure. By combining such data with advanced statistical techniques, we were able to address many criticisms of spatial studies. Cancer cases were ascertained from a registry and cancer types were studied separately. Point-based data from a region were used, avoiding aggregation within arbitrary political boundaries. Controls provided an estimate of the underlying, non-uniform population density. Our analyses controlled for many covariates not available using registry data alone. Residential history information allowed us to take latency into account, potentially quite important for diseases like cancer. However, there were only sufficient numbers of cases to perform a latency analysis for bladder cancer. Had there been a larger number of cases, the residential histories would have allowed for space-time analyses using GAMs. In a prior study, we successfully illustrated the ability to generate hypotheses for location and timing of exposures using breast cancer data [25].

Our results have a number of potential limitations. While areas of increased or decreased risk may theoretically be caused by non-uniform control selection, sampling of controls within the study area did not depend on geography. Use of residential history allows analysis of latency (when sample size is sufficient), but it produces multiple residences, a potential source of bias. Because residences were analyzed, an apparent cluster may be caused by a few diseased people moving within a small area. To examine the effect of multiple residences, our analyses were restricted to residences of longest duration. The spatial pattern of risk was similar for bladder and kidney cancers, with little difference in the location and magnitude of hot and cold spots, but the map of pancreatic cancer differed when we restricted to residence of longest duration. This suggests that the inclusion of multiple residences did not bias the bladder and kidney cancers analyses but there may be a bias in the pancreatic cancer analysis. Improved methods for analyzing data with multiple residences are needed; weighting by residence time has been suggested [26].

We computed global and pointwise p-values, but many epidemiologists prefer confidence intervals when evaluating the precision of point estimates [27]. It should be possible to compute variance bands (also known as confidence bands) for our maps [13]. We performed permutation tests that conditioned on the span size of the observed data which may be smaller than the span size for a permuted dataset under the null hypothesis of a flat map. This could possibly lead to a larger deviance statistic under the null hypothesis and the global permutation p-value would then be too large (conservative). The effect of conditioning on the original span size is a topic of future research. Pointwise tests were only conducted if the global deviance test indicated that the map was unlikely to be flat, but performing multiple testing at each location may result in an increase in the type I error rate. Although spatial analyses are useful for generating new hypotheses, the location of significant hot and cold spots should be considered exploratory.

## Conclusion

Using generalized additive models and GIS, we generated maps of bladder, kidney, and pancreatic cancer risk. When available, population-based case-control studies provide extensive data on potential risk factors and residential histories that address many methodological criticisms of cluster studies. The results of the current analysis illustrate the usefulness of GAM methods in generating hypotheses for further investigation. We identified a significant hot spot of bladder cancer that coincides with groundwater plumes. Groundwater in the study area is currently being modeled to explore this possible association between drinking water contamination and cancer.

## Abbreviations

AIC: Akaike's Information Criterion; GAM: generalized additive model; GIS: geographical information systems; MMR: Massachusetts Military Reservation; OR: odds ratio

## Competing interests

The authors declare that they have no competing interests.

## Authors' contributions

VV conducted the spatial analyses and drafted the manuscript. TW collaborated on all analytical and editorial decisions. JW provided statistical support and consulted on analytical and editorial issues. AA provided the data and assisted in epidemiologic analysis and editing. All authors read and approved the final manuscript.
